# Empowerment in Obesity and Behavioral Weight‐Management Programmes: A Scoping Review

**DOI:** 10.1111/obr.70141

**Published:** 2026-04-26

**Authors:** Corinne Bolland, Anna Robins, Phillip Gray, Paul Sindall

**Affiliations:** ^1^ School of Health and Society University of Salford Salford UK

**Keywords:** empowerment, empowerment theory, obesity, participatory approach

## Abstract

In the UK, over 25% of adults are living with obesity, with disproportionately higher rates among Black adults (35%) and those in the most deprived areas (36%). Obesity is linked to increased comorbidity, mortality, stigma, and substantial annual health costs exceeding £11.4 billion. Behavioral weight‐management programmes are the primary intervention recommended for obesity treatment, yet they demonstrate limited effectiveness, inadequate long‐term support, a lack of personalized care and poor cultural relevance. Participatory approaches, that is, bottom‐up strategies engaging communities, are increasingly applied in public health to address social determinants and reduce disparities. Individuals supported to manage their health and access care are considered “empowered”; however, this concept remains ambiguous, lacking clear definition and consistent application. Although global policy promotes empowerment to promote individual health, including weight‐management, no review has explored its role within obesity and weight‐management programmes. This scoping review examined how empowerment has been defined and implemented across behavioral weight‐management programmes. Eleven studies were identified published between 2004 and 2023, revealing poor consensus in their theoretical foundations, definitions, and practical applications of empowerment, with no UK‐based studies. To support conceptual clarity, studies definitions and empowerment‐based processes were consolidated. Further research is needed in UK settings to help define empowerment in this context and to enable effective translation between research, practice, and policy.

**Abbreviations**
BMI: body mass index
BWMPs: behavioral weight‐management programmes
EB‐BWMPs: empowerment‐based behavioral weight‐management programmes
eGFR: estimated glomerular filtration rate
ET: empowerment theory
HbA1c: glycated hemoglobin
HSET: health self‐empowerment theory
MTM: multitheory model
PRISMA: Preferred Reporting Outcomes for Systematic Review and Meta‐Analysis
PRISMA‐ScR: Preferred Reporting Outcomes for Systematic Review and Meta‐Analysis, Extension for Scoping Reviews
T2DM: type 2 diabetes mellitus
US: United States

## 1 Introduction

In 2024, the World Health Organization reported that 890‐million adults, or 15% of the global population, were classified as living with obesity (BMI ≥ 30.0 kg·m^2^) [1]. Obesity is associated with stigma [2], an increased risk of comorbid health conditions (e.g., type 2 diabetes mellitus [T2DM], cardiovascular disease, certain cancers) and reduced all‐cause mortality [3]. In England, there has been an upward trend in obesity prevalence, from 22.6% of adults in 2015/16, to 26.2% in 2022/23, with greater prevalence among Black adults (34.8%) and those living in the areas of high deprivation (35.9%) [4]. In the UK, the estimated NHS costs of obesity‐related ill health are ~£11.4 billion annually, with wider societal costs reaching £74.3 billion [5], asserting that innovative approaches to help address obesity would be of appreciable value.

Behavioral weight‐management programmes (BWMPs) are the current front‐line treatment for adults living with obesity, comprised of behavioral strategies relating to dietary intake, physical activity and behavior change [6, 7]. Treatment guidelines recommend that BWMPs should support weight‐loss through calorie deficit and by encouraging participants to set their own personalized health‐related goals to maintain lifelong change [6, 7]. Evaluations of BWMPs demonstrate modest weight‐loss immediately following programmes (~4.0 kg from baseline at 12 weeks) [8, 9] and limited weight‐loss maintenance (only 18% of participants) at 26‐week follow‐up [9]. Qualitative studies have reported that service users felt ill‐equipped to self‐manage their weight‐loss without ongoing support [8, 10]. And non‐White participants describe poor engagement and low retention on BWMPs due to poor cultural relevance [11].

### 1.1 Participatory Approaches and Patient Empowerment

It has been argued that existing models to support behavior‐change and self‐management do not sufficiently address the social and environmental determinants of obesity and place undue responsibility on those with the lowest levels of control over health‐related decision‐making [12]. In a public health context, participatory approaches are “bottom‐up” strategies that involve the participation of service‐users and communities to facilitate a deeper understanding of personalized health needs [13], address social determinants and reduce health disparity [14, 15]. Collectively, persons who are actively involved and supported to recognize and manage their own health needs, with access to appropriate health services as required, have been described as “empowered” [16].

A recent review has emphasized that the degree of empowerment is mediated by the contextual conditions of individuals, supporting the notion that participatory approaches are required to understand the empowerment needs of different populations [17]. Participatory approaches have long been recommended for the empowerment of adults living with obesity [18] yet, to date, the obesity literature demonstrates low service‐user participation and community engagement and frequent underreporting of demographic variables, which may limit the generalizability of the evidence relating to BWMPs [19].

Those who have adopted participatory approaches report positive outcomes. In adolescent obesity, participatory approaches have led to sustainable community‐led programmes [20], contextually appropriate resources [21]. programmes built on community assets [22], and improved strategies to facilitate healthy lifestyle behaviors [23]. In adult obesity, participatory approaches have been insightful, with service‐user perspectives identified and participants rating the notion of being valued and being heard [24, 25]. Collaborations between BWMPs and communities have utilized local knowledge and assets to increase accessibility and uptake [26]. The co‐design of BWMPs with local stakeholders has led to a better fit for service users, supporting health equity and therefore increasing the potential for positive health outcomes [27].

### 1.2 Empowerment Theory

Supporting the link between participatory approaches and patient empowerment, “empowerment theory” (ET) [28, 29, 30, 31] proposes that health outcomes are largely governed by the socio‐environmental context and therefore, interventions should strive to enhance the opportunity structure of local communities and to increase people's access to appropriate resources, improving people's competency to manage their own health [28]. The effectiveness of an empowerment‐based intervention is assumed to be influenced by the contextual factors within which empowerment attempts occur, meaning that a range of programmes with a variety of styles will be required to support diverse populations [29]. For example, the way in which a programme utilizes service users in its development may influence user‐participation [32]. The degree of trust a programme places in the competency of its users to shape the intervention is key [29]. When aspects of programme design are delegated to users, traditional hierarchical structures are challenged [28], creating the conditions in which empowerment is more likely to occur [30]. Central to empowerment theory is a strengths‐based perspective, which reframes users not in terms of their constraints but through recognition of their existing skills, resources, and capabilities [28]. Active user participation is a key mechanism through which empowerment is enacted: it enhances trust in users' competence to shape programmes [29], participate in decision‐making, and exercise meaningful control [30]. Programmes that also cultivate a sense of community, through social support, shared responsibility, and reciprocal helping, further shape how service users perceive themselves and one another, reinforcing the social and psychological conditions that are necessary for empowerment [29, 30].

The defining features of empowerment are presented as “perceived self‐efficacy and control.” “motivations to exert control.” and “participatory behaviors” across interdependent levels of society (i.e., at the individual, community, organizational levels) [32]. Additionally, three morally‐laden values guide empowerment in research and intervention; “distributive justice” advocating for the equitable distribution of resource; “collaborative and democratic participation.” which assumes the capacity of service users to participate in the decisions affecting their welfare, and “self‐determination” supporting the ability of service‐users to make their own decisions and to determine their own actions that are aligned with self‐identified goals, needs, and values [31]. Under these assumptions, empowerment research and intervention should be centered upon co‐production and community‐oriented approaches that are aligned with the study of individuals within their own context [33]. For the purposes of this review, Figure 1 below has been created to serve as a conceptual model of the key features of ET.

**FIGURE 1:**
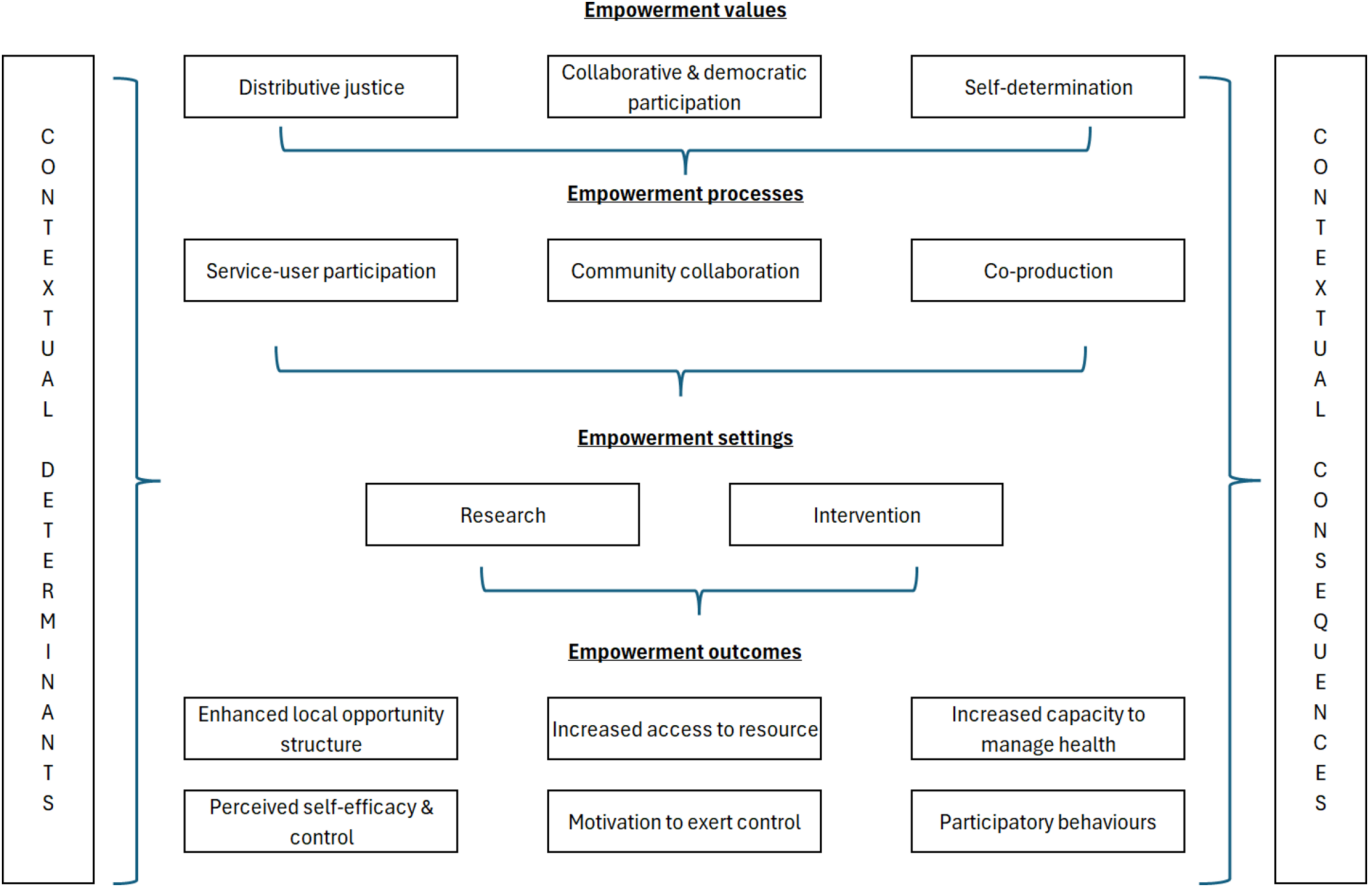
Key features of empowerment theory. A conceptual model presenting the values that guide empowerment theory, the processes through which to achieve empowerment, the settings where empowerment may take place and the outcomes of empowerment attempts. The boxes to the far left and right of the model relate to the influence of context on empowerment efforts and their consequences.

Empirical studies demonstrate that empowerment is supported through active participation in the treatment process leading to improved self‐confidence, autonomy and motivation, and greater engagement in health behaviors and disease self‐management [34]. In diabetes education programmes, empowerment approaches have led to improvements in perceived self‐efficacy for self‐management, actual self‐management behaviors, and improved health outcomes [35]. Recently, an empowerment approach has been considered as well‐suited to support behavior change in vulnerable adults [36] and immigrant women living with diabetes, hypertension, or obesity [37].

Although these observations are noteworthy, current understandings of empowerment, within health settings, are varied and complex [38, 39, 40] and provide little consensus on a definition of empowerment and show inconsistent use of ET to guide study design and intervention [41, 42, 43]. For these reasons, no conclusive evidence exists to define and explain the role of empowerment in an adult obesity context, including knowledge of the specific empowerment‐related strategies used by BWMPs to achieve and/or increase empowerment in their participants.

### 1.3 Research Question/Aim

The aim of this study was to review the literature to understand: (i) how BWMPs described as empowerment‐based (EB‐BWMPs) have understood the concept of empowerment (i.e., how did EB‐BWMPs define empowerment); (ii) the use of empowerment‐related theory to guide EB‐BWMPs; (iii) the methods and processes involved in empowerment‐based practice in adults living with obesity; and (iv) what outcomes have been reported by EB‐BWMPs to demonstrate their effectiveness.

Study objectives were as follows:

1. Identify and examine literature, which has used the term empowerment to describe their BWMP delivered to adults living with obesity;
2. Present studies definitions of empowerment and discuss their use of empowerment‐related theory;
3. Report the delivery methods chosen and inventory the empowerment‐based processes employed by EB‐BWMPs.
4. Summarize the outcomes reported by EB‐BWMPs including any attempts to operationalize and measure empowerment:

## 2 Methods

Because of the potential for considerable inter‐study heterogeneity in terms of study design, a scoping review was selected as the most appropriate means to capture, collate and express the available literature. Unlike systematic reviews, which offer synthesis and critical appraisal of the available evidence, scoping reviews enable identification and clarification of the key concepts and contextual definitions [44], and in this case offering the opportunity to inventory the empowerment‐based processes used by EB‐BWMPs while maintaining a rigorous approach [44].

### 2.1 Protocol and Registration

This scoping review adhered to the PRISMA extension for scoping reviews (PRISMA‐ScR) [45] and followed the Arksey and O'Malley five‐stage methodological framework [46]. Table 1 highlights each of the framework stages that have been applied to support the researcher to engage with the research process reflexively and ensure a comprehensive and unlimiting approach to discovering appropriate studies [46]. Identification of the research question (i.e., framework stage 1) was addressed previously and can be found in the aforementioned aims section.

**TABLE 1:** Arksey and O'Malley (2005) methodological framework.

Framework stage (FS)	Action
1	Identifying research question
2	Identifying relevant studies
3	Study selection
4	Charting the data
5	Collating, summarizing and reporting results
6 (optional)	Consultation exercise

*Note:* The optional sixth stage was not used within this review as it was deemed unnecessary to answer the research questions.

### 2.2 FS2—Identification of Relevant Studies

#### 2.2.1 Eligibility Criteria

Peer‐reviewed studies published in any year were included if they described the delivery of an EB‐BWMP, regardless of study design. Populations were limited to adults (age ≥ 18 years) who were living with obesity (BMI ≥ 30 kg·m^2^, adjusted for ethnic groups). For the purposes of time and due to difficulty in appropriately assessing the quality of unpublished reports, gray literature was excluded from this review [47]. Studies were also excluded if not written in English, involved children or pregnant people, or included nonbehavioral‐based treatment strategies (e.g., pharmaceutical or surgical procedures).

#### 2.2.2 Sources of Evidence

Databases, “CINAHL.” “Web of Science.” “Scopus.” “PubMEd.” “PsychNET.” and “Google Scholar” were searched between April 22, 2024, and April 23, 2024, with the following search terms: “Empowerment” [AND] “Obesity” [OR] “Obese” [OR] “Weight Management” [OR] “Weight‐loss.” Truncations to both empower* and obese* were made to maximize returned results where possible. A date range was not applied to searches so that all relevant studies were included. The screening process for the retrieval of studies is presented in Figure 2, including the reasons for exclusion.

**FIGURE 2:**
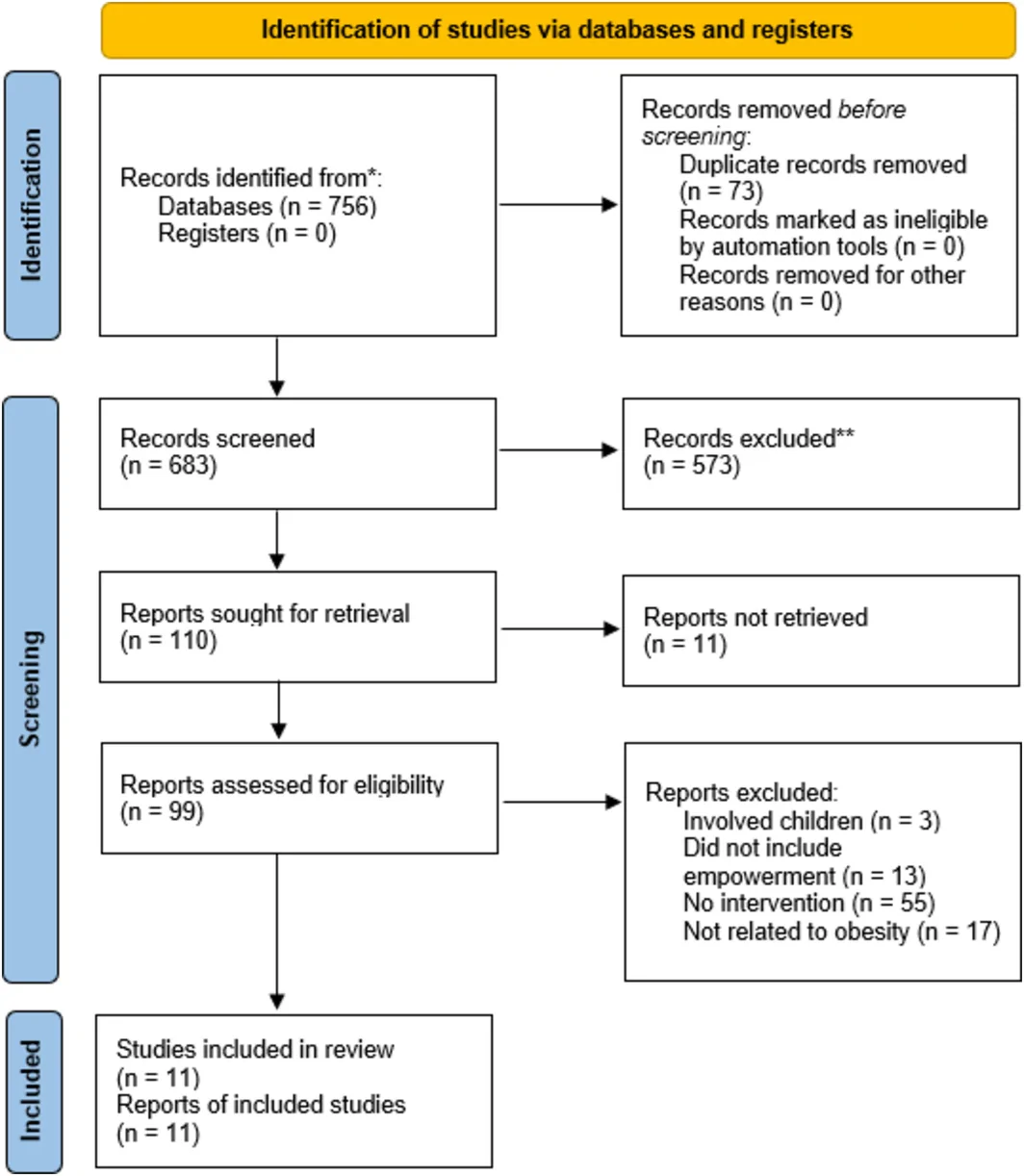
PRISMA‐ScR FLOW DIAGRAM. Diagram illustrates the selection process of evidence included in the scoping review, outlining the number of records identified, screened and assessed for eligibility with reasons for exclusion at each stage documented.

### 2.3 FS3—Study Selection

Search terms returned 756 results, and after the removal of duplicates (*n* = 73), 683 titles remained for title screening. Titles were retained if they mentioned “empowerment” and/or “obesity” and irrelevant titles (i.e., not related to the topic of empowerment) were excluded (*n* = 573). One hundred ten papers were sought for retrieval for full review, within which 11 titles were unavailable (*n* = 99 retained). Subsequent full‐text screening resulted in exclusion of 88‐ineligible studies (included children, *n* = 3; non EB‐BWMP, *n* = 13; no intervention reported, *n* = 55; did not involve adults living with obesity, *n* = 17). Therefore, 11 studies met the inclusion criteria, with reference lists screened and studies retained for full review.

### 2.4 FS4—Charting the Data

Eleven studies published between the years of 2004 and 2023 were retained for full review. The principal investigator was solely responsible for the data charting process. A summary of the characteristics of included studies was devised (Table 2), in chronological order of publication date, where charting included the sampled population, study design and reported benefits. Two tables (Tables 3 and 4) were then composed to identify the proposed definition of empowerment and application, or absence, of empowerment‐related theory and/or model (Table 3), an inventory of empowerment‐based processes adopted by EB‐BWMPs (Table 4), and an additional chart to indicate the range of empowerment‐based processes and their hierarchical frequency (i.e., the number of studies adopting each approach) (Figure 3).

**TABLE 2:** Collated characteristics of empowerment‐based behavioral weight‐management programmes.

Author (year), country, design, study duration	Study population, mean BMI/weight (SD), mean age (SD), % female, ethnicity, education, financial status	Intervention characteristics (*n*), comparator condition (*n*)	Reported outcomes of empowerment approach, important findings
Mayer‐Davis et al. (2004), USA, RCT, 12 months	Rural population with T2DM, age 60 years (no SD), BMI 36.7 kg·m^2^ (no SD), > 80% female, ethnicity 82% Black, 48% ≤ high school education	“EMPOWER” (*n* = 49): Culturally sensitive content, 16 × weekly, 4 × bi‐weekly, 6 × monthly	“EMPOWER” lost sig. more weight than “standard care” at 6 months (2.6 kg vs. 0.4 kg, *p* ≤ 0.01) and 12 months (2.2 kg vs. 0.3 kg, *p* = 0.55). HbA1c sig. reduced in all groups from baseline: “EMPOWER” (*p* < 0.01), “reimbursed” (*p* < 0.01), “standard care” (*p* = 0.05)
		“Reimbursed vare” (*n* = 47): 4 × nutrition sessions and printed resources	
		“Standard care” (*n* = 56): 1 × nutrition session and printed resources	
Geller and Crowley (2009), USA, pilot study, 2 years	Adults with developmental delay, weight 173.1 (35.2) lb, age 42.6 years (no SD), 58% female	“FUNK” Group programme (*N* = 43): Activities to simulate decision‐making, achieving success, meal preparation, personal health, 2 × weekly in year one, 1 × weekly in year two	Those attending 2 × weekly lost more weight than 1 × weekly (5.7 lb vs. 0.8 lb; no *p* value)
Amirrood et al. (2013), Iran, quasi‐experimental, 2 months	Married females, BMI 29.3 (3.6) kg·m^2^ adjusted for ethnic group, age 33.63 (8.75) years, 100% female, 43.6% had < primary education, 7.7% employed	“Empowerment programme” (EP) (*n* = 45): Group focus: Perception of threat, problem solving, nutritional education and evaluation, pamphlet shared with family members, 6 × 45‐min group sessions	Post‐EP, EP sig. improved dietary behaviors (*p* ≤ 0.001) and sig. better compared with “control” (*p* ≤ 0.001). EP sig. improved awareness (*p* = 0.001), attitude (*p* = 0.001), self‐esteem (*p* = 0.001), and self‐efficacy (*p* = 0.001). “Control” = sig. increased awareness (*p* = 0.003).
		“Control” (*n* = 45): No intervention	
Tucker et al. (2014), USA, waitlist‐control, 6 weeks	Adults from low‐income households, BMI 32.7 (7.3) kg·m^2^, 72.5% female, ethnicity 39.9% White, 37.3% black, 13.8% other, 58.8% income ≤ $40,000, (48% of group was ethnic minority and income < $24,900)	“Health self‐empowerment workshop” (HSEW) (*n* = 100): Culturally sensitive content delivered by ethnically diverse team, guidebook +1 × 121, 3 × 4‐h workshops.	Post‐“HSEW”: sig. higher engagement in PA (*p* ≤ 0.001), healthy eating (*p* ≤ 0.001), consumed less calories (*p* = 0.01), fat (*p* = 0.00), sugar (*p* = 0.01), added sugar (*p* = 0.00). Sig. greater motivation to eat healthy food (*p* = 0.00), and drinks (*p* = 0.03). Sig. less barriers to eating healthy breakfast (*p* = 0.04), healthy food (*p* = 0.03), drinks (*p* = 0.02). Sig. between group difference in BMI post‐“HSEW” (“HSEW”: BMI 31.55 (6.98) kg·m^2^ vs. “control”: 33.15 (7.37) kg·m^2^, *p* < 0.01)
		“Control” (*n* = 53): guidebook provided	
Wong et al. (2015), Hong Kong, longitudinal, 7‐sessions	Adults with T2DM, BMI 30.46 (2.90) kg·m^2^, age 61.6 (10.64) years, 61.7% female, 46.3% ≥ secondary education	“Patient empowerment programme” (PEP) (*n* = 3186): Structured education delivered by community‐based facilitators to promote knowledge, skills, self‐awareness of disease for autonomous self‐regulation, 7 × 2‐h sessions	Sig. reduced all‐cause mortality post‐“PEP”: (“PEP”/“control” = 34/59 deaths, hazard ratio (HR): 0.754, 95% CI = 0.616–0.921, *p* = 0.01). Sig. reduced rate of first composite macro‐ and microvascular event: (“PEP”/“control” = 172/229, HR: 0.580, 95% CI = 0.374–0.901, *p* = 0.02).
		“Control” (*n* = 3186): Propensity‐matched data	
Hopper et al. (2017), USA, quasi‐experimental, 6 months	Women in counties with low health expectancy, BMI 32.79 (0.90) kg·m^2^, age 54.7 (1.6) years, 100% female, 82% African American or American Indian, 32.2% high school graduate, 30% income ≤ $10,000, 39% employed	“Health, Opportunities, Partnerships, Empowerment” “HOPE” (*N* = 236, *n* = 146@6 months): Community based participatory research (CBPR) framework, led by community members, working with a Community action council, core topics adapted in line with community needs. Choice of delivery schedule equal to 4× hours per month × 6 months	Sig. weight loss 3.33 lb (95% CI = 5.40–1.27, *p* = 0.003), sig. reduction in BMI 0.56 kg·m^2^ (95% CI = 0.92–0.20, *p* = 0.005), and sig. increase in fruit consumption 0.21 point (95% CI = 0.01–0.41, *p* = 0.04)
Tucker et al. (2017), USA, waitlist‐control, 6 weeks	Churchgoers in urban New York, BMI 34.5 kg·m^2^, 81.4% female, 100% African American, 34.3% high school education, 28.6% income < $20,000	“Church‐based health smart programme” (HSP) (*n* = 37): Expert discussions and Q & A, group exercise, health empowerment coaches recruited from local churches, 1 × 121 session followed by 4 × 90‐min sessions	Compared to “control.” “HSP” sig. increased healthy eating (+0.26 vs. + 0.03, *p* = 0.001), physical activity (+0.56 vs. + 0.13, *p* = 0.000), and lost sig. more weight (lb) at programme‐end (*p* = 0.009) and compared to “control” (−2.82 lb vs. − 0.43 lb, *p* = 0.05).
		“Control” (*n* = 33): intervention poststudy period	
Muscogiori et al. (2019), Italy, pilot study, 4 months	Residents of Naples district with prediabetes, BMI 32.23 (6.08) kg·m^2^, age 60.5 years, 50% female	“Empowerment group” (*N* = 42): Fortnightly education sessions on the Mediterranean diet delivered by nutritionists	Sig. improvements from baseline in BMI (32.23 [6.08] vs. 30.60 [5.79] kg·m^2^), HbA1c % (6.30 vs. 5.85%), waist circumference (104.55 vs. 100.0 cm), SBP (130.00 vs. 120.00 mmHg), DBP (85.00 vs. 70.00 mmHg), total cholesterol (187.62 (42.63) vs. 174.76 (39.21) mg/dL), HDL (45 vs. 51 mg/dL), LDL (107.97 [37.43] vs. 94.45 [33.49 mg/dL]) (all *p* < 0.001), and visceral adiposity index score (5.86 vs. 4.18; *p* = 0.001).
Onyegbule et al. (2021), USA, quasi‐experimental time series, 12 months	African American women, BMI 37.4 (5.2) kg·m^2^, age 35.75 (no SD) years, 100% female, 100% African American, 25% had graduate degree	“Health belief and empowerment model” (*N* = 28): Stakeholder involvement in implementation, barriers identified and resolved through community collaborations, feedback from focus groups for cultural appropriateness, free transport to and from sessions, free vegetable boxes, volunteer babysitting service. Evidence‐based nutrition and PA sessions 3–5 times × weekly and bi‐weekly focus groups.	Improved knowledge of good nutrition (*p* = 0.057), reduced BMI (37.4 (5.2) vs. 28.5 (4.5) kg·m^2^; no p), reduced weight (248.0 (33.0) vs. 190 (29.0) lb) and waist circumference (38.7 (2.0) vs. 33.2 (1.7) inches), minutes of physical activity (18.7 (6.0) vs. 58.6 (4.1) min), and reduced total cholesterol (241.0 (49.0) vs. 186.0 (17.0) mg/dL)
McGrane‐Minton et al. (2021), USA, quasi experimental, 7 months	Low‐income adults concerned with weight, age 51.7 (10.9) years, 92% female, 43.8% Black, 43.8% White, 20.9% high school diploma, 54.2% unemployed	“Health empowerment program” (*N* = 153): Biopsychosocial‐spiritual approach, CBPR framework, collaboration between researcher and community, use of community assets, delivery in faith‐based neighborhood health center, shared dissemination	Sig. improvements in depression (*β* = −1.21, SE = 0.24, *p* ≤ 0.001) and anxiety (*β* = 0.69, SE = 0.24, *p* = 0.005) over time. Sig. increase in self‐regulation (*β* = 2.42, SE = 0.82, *p* = 0.003). Sig. increase in fruit and vegetable consumption (*β* = 0.78, SE = 0.22, *p* ≤ 0.001), and food quality (*β* = 0.97, SE = 0.16, *p* ≤ 0.001).
Joveini et al. (2023), Iran, quasi‐experimental, 5 weeks	Middle‐aged women with high sugar consumption, BMI in empowerment programme: 33.22 (4.96); in control: 43.12 (3.88) kg·m^2^, age 33.7 (no SD) years, 100% female	“Empowerment programme” (EP) (*n* = 58): use of “multitheory model” (MTM), 5 × 60‐min sessions to improve knowledge, self‐efficacy	Sig. effect of EP on weight and BMI (both *p* ≤ 0.001). Sig. lower BMI (*p* ≤ 0.001), weight (*p* = 0.023), and waist circumference (*p* = 0.010) at 6 months. Self‐reported sugar consumption sig. lower in BWMP versus control at 1 (*p* = 0.018), 3 (*p* = 0.002), and 6 months (*p* = 0.001).
		“Control” (*n* = 57): No intervention	

**TABLE 3:** Definitions of empowerment and application of empowerment (or related) theory and/or model.

Author (year)	Definition of empowerment (Y/N)	Empowerment theory (or empowerment‐related) and/or model used (Y/N)
Mayer‐Davis et al. (2004)	N	N
Geller and Crowley (2009)	N	Y = “FUNK” model: adapted for adults with developmental delay. Emulating empowerment through group participation and making choices
Amirrood et al. (2013)	“Empowerment is taking an active role in the care of one's own health.”	Y = “family empowerment model” (Alhani et al. 2003): change is dependent on the degree of motivation, cognitive processes (e.g., self‐esteem, self‐control, self‐efficacy) and personality traits (e.g., knowledge, attitude, perceived threat)
Tucker et al. (2014)	Empowerment is having control over one's health under whatever conditions exist	Y = “health self‐empowerment theory” (Tucker 1999): Social injustice and inequalities contribute to the prevalence of obesity. Inequalities are intractable and therefore empowerment is to seek control over health under existing conditions
		Community‐based “participatory research” (CBPR): utilizing the existing strengths and assets of communities and advocating for communities to take action
Wong et al. (2016)	Empowerment is the knowledge, skills, and self‐awareness of own disease to promote autonomous self‐regulation to maximize health and wellbeing	N
Tucker et al. (2017)	See Tucker et al. (2014)	Y = see Tucker et al. (2014)
Hopper et al. (2017)	N	CBPR
Muscogiuri et al. (2019)	Empowerment is increased knowledge concerning self‐care and possessing the skills, self‐awareness and a sense of personal autonomy	N
McGrain‐Minton et al. (2019)	N	CBPR
Onyegbule et al. (2019)	Empowerment is marginalized communities who come together and utilize their strengths and resources to ensure healthy, active and improved quality of life	Y = “empowerment model” (Moran 2017): addressing perceived and actual barriers to behavior change. Strategies to support learning and skills development. Collaborative conversations producing community‐led solutions
Joveini et al. (2023)	N	Y = “multitheory model” (Nahar et al. 2016): Onset of health behavior through participatory dialogs, behavioral confidence, and changes to physical environment. Maintenance of health behavior through emotional transition, resource availability, and social environment

**TABLE 4:** Inventory of empowerment‐based processes.

**Self‐determination**—“an ability to make own decisions and determine own actions aligned with self‐identified goals, needs and values”	**Collaborative and democratic participation**—“an assumed capacity of individuals to participate in decisions affecting their welfare”	**Distributive justice** —“the equitable allocation of resource”
**Knowledge and skill development**	**Collaboration (with community)**	**Advocacy**
Sharing health condition‐specific information	Community Based Participatory Research (CBPR)	Review of local resources, strengths and needs
Health education provided to other family members	Community members having active roles in all aspects of	Transport provided
Health panel Q and A sessions	Development, delivery, and research	Responding to established group/individual needs
Sharing tips on self‐management/maintenance	Establishing community identity and needs	
Problem solving activities	Community Action Councils	
Guidance on financial literacy	Collaboration with community venues and their staff	
Budgeting strategies	Programme topics enhanced through regular meetings	
Emotion regulation/stress management	Review of programme fidelity and relevance through feedback	
Cooking demonstrations		
**Awareness (and increased access)**	**Collaboration (with others)**	**Utilization of strengths and assets**
Simplification of written materials and content	Participatory group dialog	Delivery in community‐based venues
Reading assistance as required	Group brainstorming	Cultural/specific adaptations to programme content
Sessions provided in other common languages	Group sharing of own health behaviors and strategies	And implementation based on participant feedback
Adaptation/simplification of self‐monitoring tools	To overcome barriers	Community‐based staff and volunteers
Culturally appropriate/representative content and materials	Group discussions on pros and cons of changing behaviors	Community members trained to deliver programmes
		Fruit and vegetables donated by local organization and
Culturally tailored activates	Developing a sense of community through group work	Delivered to participant homes
	Use of focus groups to evaluate EB‐BWMP	Volunteer babysitting service during sessions
**Autonomy**	**Collaboration (with healthcare practitioners)**	
Participant choice of session time/frequency	Customized workshop activates based on reported	
Post‐programme follow‐up session	Barriers and motivations	
Activities to simulate making choices	Mutual involvement in goal‐setting sessions resulting in treatment plans	
Session content to support feelings of success		
Personalized goal setting	Participatory dialog with programme leaders	
Client‐led strategies to achieve goals	Programme adaptations following participant feedback	
Role play	Opportunities to ask programme leaders health‐related questions	

*Note:* Empowerment‐based processes are categorized against the three values of empowerment theory. Each column contains subcategories based on the empowerment themes derived by this review.

**FIGURE 3:**
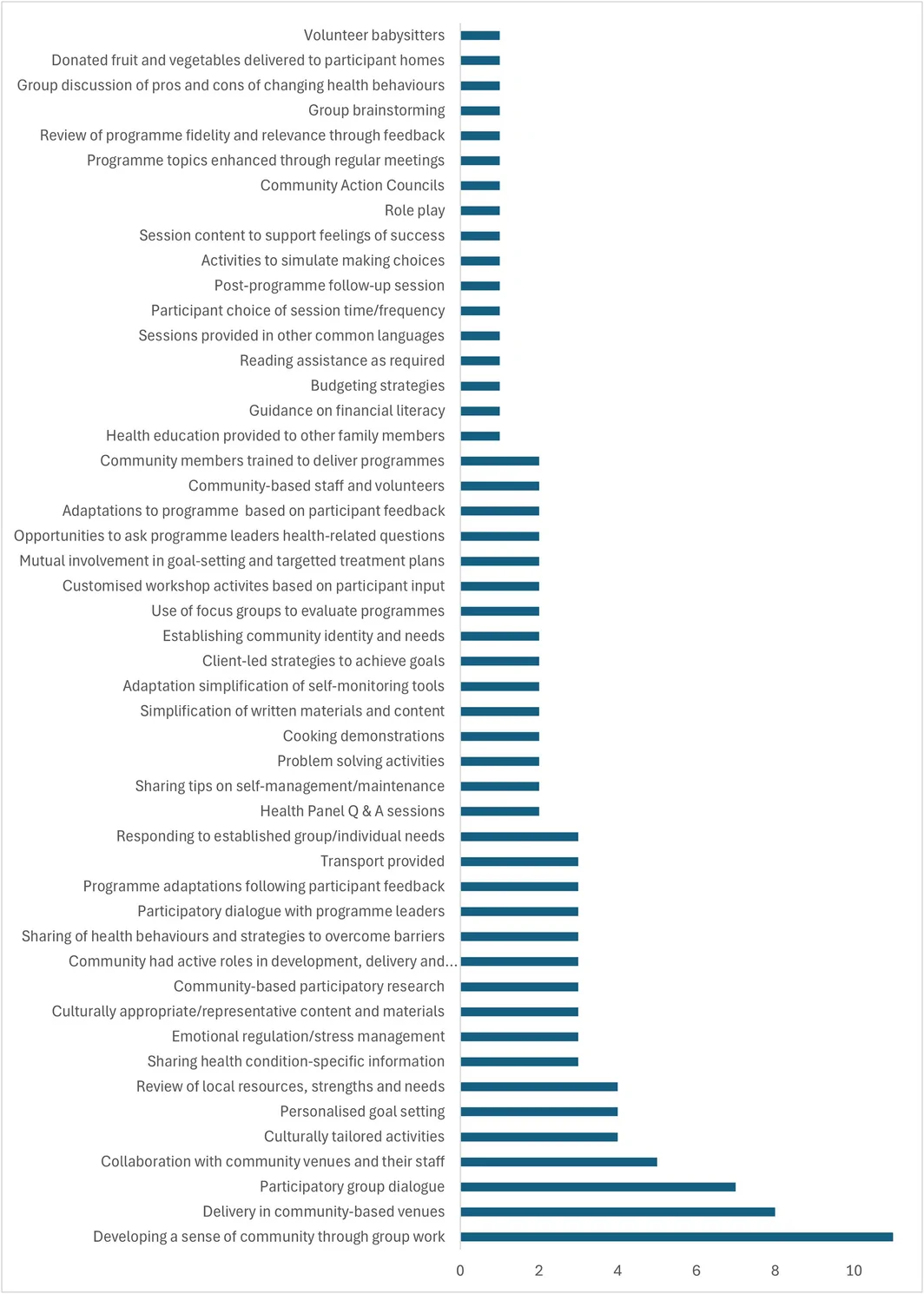
Empowerment‐based processes. Processes are presented hierarchically to demonstrate the frequency with which they were adopted by the included studies (x‐axes: number of studies including each process).

### 2.5 FS5—Collating, Summarizing, and Reporting Results

#### 2.5.1 Study Design

Eleven studies described themselves as delivering an EB‐BWMP in populations living with obesity. One study had a longitudinal design [48], one was a randomized controlled trial [49], two had a waitlist‐control design [50, 51], two were pilot studies [52, 53], and five adopted a quasi‐experimental, pretest posttest design [54, 55, 56, 57, 58].

#### 2.5.2 Demographics

No studies were found in a UK setting. Studies were located in the United States (*n* = 7), Iran (*n* = 2), Hong Kong (*n* = 1), and Italy (*n* = 1). Considerable variability in sample size was observed (range = 28–6372 participants). Participants were mostly female (*n* = 4749, 64.6%). The mean age of participants ranged from 33.6 to 61.6 years and mean BMI ranged from 29.3 to 37.4 kg·m^2^.

Ethnicity was reported by seven studies, with participants (*n* = 744) identifying as African American (*n* = 417, 56%), White (*n* = 204, 27%), Hispanic/Latino (2 studies, *n* = 25, 3%), Asian (2 studies, *n* = 14, 2%), “other” (3 studies, *n* = 11, 1%), and biracial (1 study, *n* = 3, 0.4%). Participant income was reported by only four studies (*n* = 522) with 72% of participants (*n* = 376) reporting an annual income of ≤ $40,000.00 US dollars. Level of education was reported by eight studies (*n* = 7114), with 44% of participants described as having less than, or equal to, a high school education (*n* = 3149) and 5% (*n* = 362) reporting higher education.

#### 2.5.3 Populations

Four studies had culturally diverse samples [49, 50, 55, 57]. Two studies specifically recruited African American females [51, 58], and two recruited married Iranian females [54, 56], two studies targeted adults with T2DM [48, 49], and one sampled adults with developmental delay [52].

#### 2.5.4 Definitions of Empowerment

When discussing empowerment, only three studies explained the term consistently throughout [50, 51, 54]. One appeared to use the term interchangeably with patient knowledge and education [53] and one with motivation to engage in health behaviors [58]. One mentioned empowerment only within the constraints of the name of their EB‐BWMP [52].

As shown in Table 3, five studies made no attempt to define empowerment [49, 52, 55, 56, 57]. Of the six studies who did provide a definition or interpretation of empowerment, similarities in the definitions adopted were observed but a uniform definition was not proposed nor complete consistency evident between and within geographical locations. Themes across definitions were related to “knowledge, skills and self‐awareness” [48, 53], “taking an active role.” [54] and “control and autonomy.” [48, 50, 51, 53] Some definitions referred to influence of context and included the use of community resource [58] and the ability to maintain health under whatever conditions exist [50, 51].

#### 2.5.5 Use of Empowerment‐Related Theory

Interestingly, not one single study utilized ET in the format presented by this scoping review. Four studies [50, 51, 55, 57] applied the principles of community‐based participatory research (CBPR), which centered firmly on utilizing the existing strengths and assets of communities and advocating for communities to take action.

Four different empowerment models were delivered and described by EB‐BWMPs. An “Empowerment Group Model” (described as a “FUNK” model) was adapted for adults with developmental delay with sessions designed to emulate empowerment through group participation and making choices [52]. Another “Empowerment Model” [59] delivered to African American females addressed both perceived and actual barriers to behavior change through embedded strategies to support learning and skills development and the active encouragement of participants to collaborate in conversations leading to community‐led solutions [58]. A “Family Empowerment Model” was employed to empower families to engage in health behaviors through the empowerment of women [54]. In this model, empowerment was supported through increasing awareness (i.e., knowledge) and problem‐solving skills to increase participants self‐efficacy for behavior change [54]. A “multitheory model” (MTM) [60] focused on participatory dialog to increase behavioral confidence to initiate health behaviors and aimed to promote the maintenance of health behaviors by addressing topics such as emotional transformation, resource availability, and changes to the social environment [56].

Two studies utilized “health self‐empowerment theory” (HSET) to guide EB‐BWMPs delivered to adults in culturally diverse, low‐income, [50] and Black populations. [51] HSET asserts that the social injustices and inequalities experienced by disadvantaged groups are intractable, and therefore, contribute to the high prevalence of obesity. HSET presents five cognitive and empowerment‐oriented, health promoting variables to be achieved through participatory approaches within communities: “health motivation”; “health self‐efficacy”; “self‐praise of health‐promoting behaviors”; “active coping styles” (i.e., skills to manage emotions); and “health responsibility and knowledge.” [61].

In order to synthesize current knowledge, and provide much needed clarity, six broad conceptual themes were delineated from the definitions and empowerment‐related theories and models presented by EB‐BWMPs. These were as follows: “advocacy”; “awareness”; “collaboration”; “knowledge and skills development”; “utilization of strengths and assets”; and “autonomy.” Based on these themes, the present study has developed a novel definition of empowerment, as the concept relates to EB‐BWMPs (Figure 4.):

**FIGURE 4:**
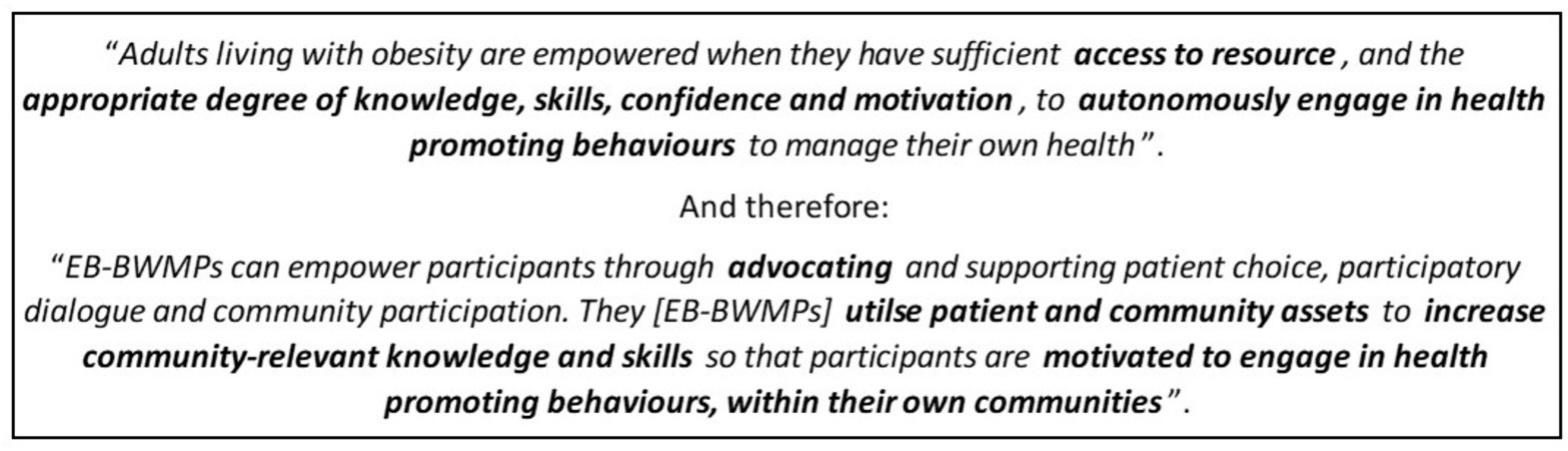
Contextually‐specific definition of empowerment. The included studies' definitions of empowerment and empowerment‐related theory were consolidated to develop a definition of empowerment in the context of obesity and empowerment‐based behavioral weight‐management programmes.

To continue to align with ET, the six conceptual themes were added in blue to the conceptual model previously presented in Figure 1. This updated model is presented below in Figure 5.

**FIGURE 5:**
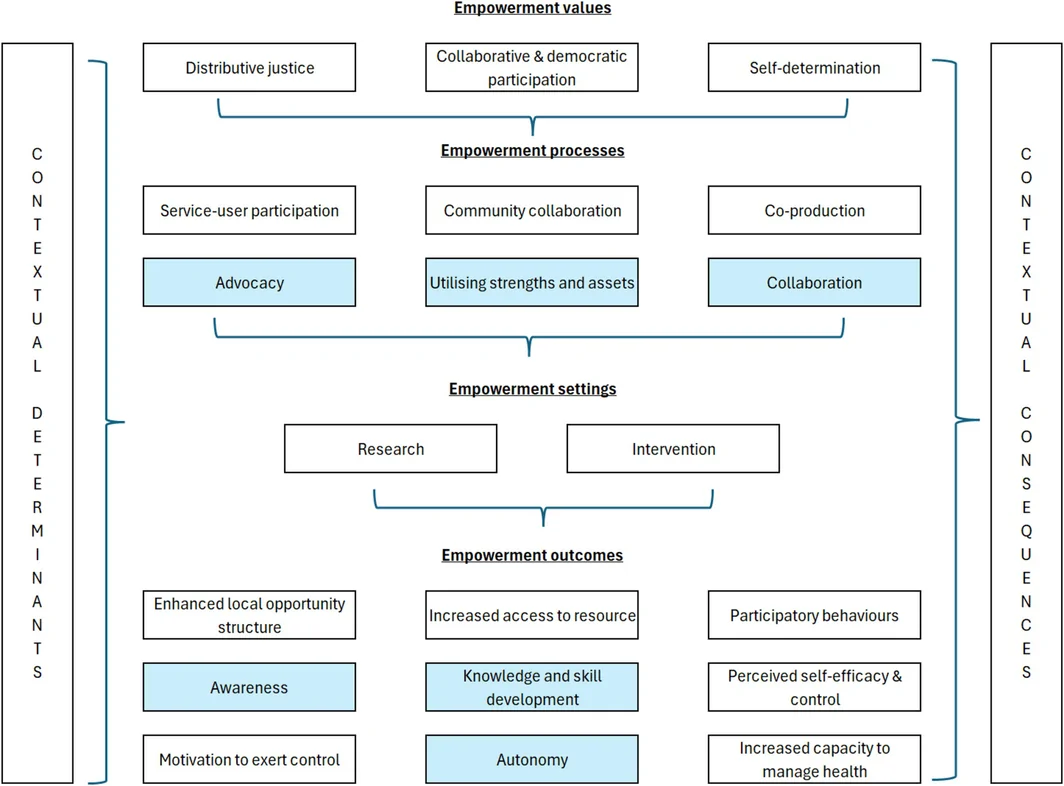
Conceptual model of empowerment in obesity and empowerment‐based behavioral weight‐management programmes. Building on the conceptual model in Figure 1, the six empowerment themes have been added (blue boxes) to increase the models contextual specificity to adults living with obesity and behavioral weight‐management programmes.

#### 2.5.6 Delivery Methods

There was notable heterogeneity across EB‐BWMPs duration (range = 6 weeks to 2 years), number of sessions (range = 4–156 sessions), number of delivery hours (range = 4.5–156 h), and choice of programme leader (clinical professional and/or community member). There was consistency across all EB‐BWMPs in that they were delivered in face‐to‐face groups or workshop settings mostly within community venues or local healthcare centers familiar with study participants.

#### 2.5.7 Empowerment‐Based Processes

In total, 49 unique empowerment‐based processes were reported by EB‐BWMPs. The number of processes adopted by individual EB‐BWMPs varied broadly. One study incorporated 19 processes into their EB‐BWMP [50], one 18 processes [51], and two 16 processes [49, 57]. One study applied 12 processes [55]. Four studies each delivered a total of 8 processes, [48, 52, 56, 58] one 5 processes, [54] and one 2 processes [53].

Again, to ensure the current findings were aligned with ET, empowerment‐based processes were categorized against each of the three values of ET: “self‐determination”; “distributive justice”; and “collaborative and democratic participation” (see Table 4). In this table, further subcategories were added to represent each of the conceptual themes identified by this review. For example, the value of “self‐determination” has the following three subcategories: “knowledge and skill development.” “autonomy.” and “awareness” with each relating to a specific set of processes for research and intervention.

As shown in Figure 3, empowerment‐based processes were also presented hierarchically to demonstrate the frequency with which they were described by EB‐BWMPs. Developing a sense of community through group work was the most commonly employed empowerment‐based process and was adopted by all 11 EB‐BWMPs. The second most common was delivery in a community‐based venue, which occurred in eight (73%) of the EB‐BWMPs. The use of participatory group dialog was described by seven (64%) EB‐BWMPs. Collaboration with community venues and their staff was described five times (45%). The remaining 45 processes were each described by EB‐BWMPs less than five times.

### 2.6 Reported Outcomes of Empowerment‐Based Behavioral Weight‐Management Programmes

#### 2.6.1 Health Outcomes

As detailed previously in Table 2, the reported outcomes following EB‐BWMPs were mostly unrelated to the construct of empowerment and not surprisingly related to participant health (weight and/or BMI, blood pressure, and waist circumference), including metabolic markers (HbA1c, lipid profile, fasting glucose, eGFR, and occurrence of micro/macrovascular event), engagement in health behaviors (dietary intake and physical activity), and psychological variables (depression, anxiety, and stress). Certain cognitive variables (e.g., motivation, self‐efficacy, goal‐orientation, and self‐regulation) that were somewhat related to the working definition of empowerment were included, but were not chosen by studies to represent empowerment.

#### 2.6.2 Empowerment‐Based Outcomes

Two studies developed measurement tools related to their chosen empowerment models [54, 56]. The “Empowerment Evaluation Questionnaire” (37 items) [54] measured empowerment across the following four domains: “awareness.” “attitude.” “self‐esteem.” and “self‐efficacy in dietary behaviors” (utilizing “Rosenburg's Self‐Esteem Scale”) [62]. Domain scores ranged between 0 and 25 to reflect the degree of change over time. Scores across domains were aggregated (ranging from 0 to 100) and banded to reflect the overall degree of empowerment (scores: 0 to 33.0 = “weak.” 33.1 to 66.0 = “average.” and 66.1 to 100 = “good”). Content validity was assessed by a panel of 10 health education specialists. Cronbach's alpha revealed acceptable internal consistency for the composite measure (*α* = 0.78), and acceptable to good for subdomains of awareness (*α* = 0.71), attitude (*α* = 0.71), self‐esteem (*α* = 0.81), and self‐efficacy (*α* = 0.79) [54]. Following the programme, discrete paired *t*‐tests revealed significant within‐group differences for time (PRE vs. POST) in the experimental group (EMPOWER) for all subdomains (*p* < 0.001). In contrast, no significant within‐group differences were observed for control [54].

The second measurement tool related to the domains of the MTM [56]: “participatory dialog”—12 items; “behavioral confidence”—9 items; “changes in physical environment”—6 items; “emotional transformation”—6 items; “practice for change”—4 items; “changes to social environment”—4 items in addition to two further items relating to “behavioral intentions” and “intention to maintain behaviors.” This study also reported significant improvements across all domains following their EB‐BWMP (*p* < 0.001) and when compared to their control group (*p* < 0.001). The authors reported that behavioral confidence had a significant impact on participants behavioral intention (*p* = 0.001) and that higher scores in the domains of practice for change and in changes to the social environment were significantly associated with behavioral maintenance (*p* = 0.013 and *p* = 0.006, respectively). The tool was reported to have face validity (qualitative review with 20 women), content validity (qualitative review with 11 health education specialists), and acceptable to excellent internal consistency: participatory dialog—pros (*α* = 0.92); participatory dialog—cons (*α* = 0.93); behavioral confidence (*α* = 0.89); “changes in the physical environment” (*α* = 0.71); emotional transformation (*α* = 0.73); practice for change (*α* = 0.80); change is social environment (*α* = 0.76); “start behavioral changes” (*α* = 0.93), “maintain or continue change” (*α* = 0.93) (50). Unfortunately, neither study presented a complete measurement tool, and thus results should be interpreted with caution.

## 3 Discussion

This scoping review is the first to offer a detailed examination of empowerment‐based practice in EB‐BWMPs delivered to adults living with obesity. The primary aim of this study was to understand how EB‐BWMPs have understood the concept of empowerment, particularly with regard to the definitions chosen to represent empowerment and the empowerment‐related theories and/or models used to guide programmes. Of the included studies, while half (55%) of EB‐BWMPs provided a definition of empowerment, there was no consensus, with an agreed definition not being uniformly proposed across studies. This finding is congruent with previous reviews of the health literature, such as Acuna Mora et al. [41] who found 35 unique definitions of empowerment; Cerezo et al. [39] who found 17 definitions, and Castro et al. [38] who found 20 definitions of empowerment, each with a different emphasis that was dependent on the study context and/or academic discipline (e.g., psychology, nursing). Campbell et al. [42] have suggested that the lack of a universal definition is reflective of a “definitionally imprecise” construct that is “inherently complex and subjective” to specific populations.

When examining the use of empowerment‐related theory, we found that no study had cited or applied ET as presented by this scoping review. Despite these chosen empowerment models/theories being inconsistent, overall they were in agreement with ET in that EB‐BWMPs sought to increase self‐efficacy and motivation to self‐manage health through participatory methods. As a consequence, it was possible for this study to present six broad conceptual themes and a contextually specific working definition of empowerment. A further strength of the present study was the introduction of a novel conceptual model of empowerment that was grounded in ET and enhanced by the findings of this review. All of which supports consensus‐building of empowerment‐based practice in adults living with obesity.

When empowerment‐based practice was described, it revealed significant heterogeneity across the delivery methods chosen and little consistency in the number of empowerment‐based processes employed by EB‐BWMPs. It was notable that of the 49 unique processes, a total of 45 were used less than 5 times. Furthermore, when processes were mapped against the values of ET (see Table 4), it was evident that the most frequently used processes were geared toward supporting collaborative and democratic participation and were less focused on developing self‐determination and distributive justice. We believe that the inventory presented by this review will be of considerable practical value in guiding the actions of existing EB‐BWMPs, toward supporting each of the values of ET, which are positioned to meet local needs.

The outcomes reported by EB‐BWMPs suggest that an empowerment approach reduced participant barriers and increased participant knowledge, motivation, and engagement in positive health behaviors. Studies also reported improved health‐related outcomes such as reduced body‐weight and weight circumference. However, due to empowerment‐based processes being used infrequently and inconsistently, and without a standardized approach, it is pertinent that further research is undertaken to establish which processes, or combinations of processes are best deployed to support participant outcomes.

With regard to the degree of participant empowerment, only two of the 11‐studies attempted to measure empowerment, with each having operationalized the construct based on their own empowerment models meaning that data could not be pooled nor compared. For example, the first tool [54] operationalized domains relating to critical consciousness (e.g., awareness, attitude, self‐esteem, and self‐efficacy) and presented each as antecedents of empowerment. In contrast, the second tool [56] measured variables related to participant actions (e.g., participatory dialog, changes to the physical environment, and practice for change) and cognitive variables relating to behavioral confidence and emotional transformation. Importantly, neither tool was presented by the authors for future use and/or validation meaning it is uncertain if the results could be replicated, or if results would have varied had a different tool been used. We believe that this finding is unsurprising considering the current lack of clarity on empowerment in this context.

What was unexpected was the absolute lack of studies conducted in a UK setting. This was especially noteworthy given the fact that current policy advocates the importance of empowerment for better self‐management of weight [15]. This may be in part due, and which may be considered a limitation of this review, to the omission of studies using terms other than empowerment to describe their EB‐BWMP. For example, the terms “patient enablement.” “patient activation.” and “patient involvement” are often used synonymously with the term empowerment [63]. However, it has been supported that empowerment is markedly distinct from its related constructs [64] strengthening the approach of the present study to avoid further ambiguity and include only studies referring explicitly to empowerment.

Given this absence in the literature, it would be prudent to validate the working definition and novel conceptual model presented by this review by engaging adults with lived experience of obesity, who should be considered key stakeholders in EB‐BWMPs. It would also be sensible to ascertain the views of other stakeholders, for example, service providers and the commissioners of BWMPs as these individuals are (a) responsible for shaping and resourcing services and (b) the working definition and model proposed within the present study involve the actions of EB‐BWMPs to facilitate empowerment. Such an approach would build on existing knowledge to further define the concept of empowerment in an obesity context, and thereby, shape UK service design and delivery.

### 3.1 Limitations

Because restraints on both time and resource, only studies published in the English language were included, which may have resulted in language bias and the exclusion of relevant research published in other languages [65]. This limitation potentially narrows the generalizability of this review [66]. Secondly, the screening of studies for eligibility was conducted by the lead researcher only, which may have introduced selection bias or increased the risk of overlooking relevant studies [67]. Although every effort was made to follow rigorous screening procedures, the absence of a second reviewer limits the reliability and reproducibility of the inclusion process [67].

## 4 Conclusions

The findings of this scoping review are in agreement with previous reviews of empowerment in broader health contexts. This study has specifically identified that a similarly inconsistent approach has been applied to the study of empowerment within an EB‐BWMP context. This lack of consensus regarding interpretations of empowerment, its intended practical application(s), use of guiding theories and/or models, and the associated processes chosen to enable and maintain empowerment compounds the issue.

This present study has provided a novel definition of empowerment, a conceptual model and an inventory of empowerment‐based processes, which have helped to provide greater clarity. However, further work is required to specifically define what constitutes empowerment from the service‐delivery and ‐user perspectives, how empowerment might be achieved, and how specific approaches might be designed and adopted to enhance obesity treatment outcomes. Therefore, a suggested next phase is to obtain a clear understanding of what empowerment means to core stakeholders, and how it might be enhanced by EB‐BWMPs.

## Conflicts of Interest

The authors declare no conflicts of interest.

## Data Availability

The dataset supporting the conclusions of this article is included within the article.
